# Aluminium neurotoxicity beyond the brain: a mechanistic systematic review of autonomic and peripheral dysfunction

**DOI:** 10.3389/fphys.2026.1891526

**Published:** 2026-09-16

**Authors:** Trina Sengupta, Tripti Sharma, Prasunpriya Nayak, Sovan Bagchi

**Affiliations:** 1 Department of Physiology, Employees' State Insurance Post Graduate Institute of Medical Sciences and Research (ESI-PGIMSR) and ESIC Medical College and Hospital, Joka, Kolkata, India; 2 Department of Physiology, All India Institute of Medical Sciences, Jodhpur, India; 3 Department of Physiology, College of Medicine, Gulf Medical University, Ajman, United Arab Emirates

**Keywords:** aluminium, autonomic nervous system, baroreflex sensitivity, blood-brain barrier, central nervous system, enteric nervous system, heavy metals, nervous system

## Abstract

Aluminium (Al) is a ubiquitous environmental neurotoxicant primarily recognized for its role in central nervous system (CNS) dysfunction. However, emerging evidence suggests that the autonomic (ANS) and peripheral (PNS) nervous systems may be susceptible to Al-induced damage earlier than the CNS, offering potential windows for early detection. This mechanistic systematic review synthesizes current human, animal, and mechanistic evidence to evaluate the multisystemic effects of Al beyond the CNS and to identify potential candidate markers of exposure. Following PRISMA guidelines, a comprehensive search was conducted across five databases (PubMed/MEDLINE, Embase, Scopus, Web of Science, and Cochrane Library). Sixteen primary studies that met strict inclusion criteria were analyzed. Given the heterogeneity of the data, a mechanistic synthesis was employed to map pathways and link them to clinical observations. Synthesis of the evidence reveals a consistent “Barrier-Stress Axis.” Mechanistically, Al targets tight junction proteins (e.g., occludin, claudin-5) in the blood-brain and blood-nerve barriers, leading to oxidative stress in the nucleus tractus solitarius and peripheral nerves. Clinically, this manifests as reduced heart rate variability (HRV) and impaired baroreflex sensitivity in both occupational human cohorts and experimental rat models. While peripheral nerve conduction changes are noted, human data remain limited by small sample sizes and confounding environmental factors. Preliminary evidence suggests that ANS and PNS dysregulation may precede overt cognitive decline in Al toxicity. HRV and baroreflex sensitivity are promising candidates for early-stage screening. However, these findings should currently be viewed as hypothesis-generating; longitudinal human studies are required to validate these markers and control for occupational co-exposures.

## 1 Introduction

Aluminium (Al) is the most abundant metal in the Earth’s crust and is commonly found in various aspects of daily life, including food additives, utensils, and cosmetics. However, it has no known physiological function in humans and is biologically reactive, potentially disrupting cellular processes, especially in the nervous system (Exley, 2013). Research on Al toxicity predominantly targets the central nervous system (CNS), linking it to cognitive impairment and Alzheimer’s disease (AD) pathology. However, the autonomic nervous system (ANS), peripheral nervous system (PNS), and enteric nervous system (ENS) also interact with the CNS, regulating key physiological functions. Disruptions in any one of these systems can affect the others through feedback mechanisms (Kawahara and Kato-Negishi, 2011).

Some studies related to AD-like pathology showed autonomic neuropathy but no significant changes in the heart rate variable HRV (Allan et al., 2005). A meta-analysis of HRV studies in AD reports conflicting evidence, with abnormalities in the time domain but not in the frequency domain (Da Silva et al., 2017). Vascular dementia caused by a stroke blocking any major cerebral artery may also be associated with ANS dysfunction, but the results are inconsistent. The common symptoms of autonomic dysfunctions related to dementia are orthostatic dizziness, syncope, falls, urinary tract symptoms, and constipation (Allan et al., 2006).

Genetic predisposition, environmental pollutants, and occupational hazards contribute to autonomic dysfunction. Prolonged exposure to chainsaw vibrations among forest workers negatively affects cardiac parasympathetic activity (Murata and Araki, 1991). Additionally, heavy metals can exacerbate environmental factors leading to autonomic dysfunction. Autonomic neurotoxicity from lead, copper, and zinc exposure was evaluated by measuring R-R interval variations on electrocardiogram (ECG), indicating that subclinical lead toxicity affects both the parasympathetic nervous system and the overall ANS (Murata and Araki, 1991).

Studies in both animals and humans suggest that Al exposure can impair cardiovascular autonomic control, induce peripheral nerve inflammation, and alter gastrointestinal function, even at dietary-equivalent doses (He et al., 2003; Martinez et al., 2018). Such changes may occur alongside or before cognitive impairment, raising the possibility that autonomic and peripheral involvement reflects early neurotoxic effects (He et al., 2003; Martinez et al., 2018).

Importantly, in other neurodegenerative conditions such as Parkinson’s disease, autonomic dysfunction has been shown to precede and independently predict overt cognitive decline, raising the question of whether a similar temporal relationship exists for Al-induced neurotoxicity (Martinez‐Nunez et al., 2026).

Recent reviews of Al neurotoxicity have predominantly centered on central nervous system and cognitive outcomes — spanning Alzheimer’s disease, autism spectrum disorder, alcohol use disorder, multiple sclerosis, Parkinson’s disease, and dialysis encephalopathy (Bryliński et al., 2023).

On pharmacological interventions against Al-induced learning and memory deficits in rodent models (Hayat et al., 2025).

This mechanistic systematic review, therefore, aims to critically synthesize human, animal, and mechanistic evidence on Al-induced autonomic and peripheral nervous system dysfunction, evaluate the strengths and limitations of existing data, and identify priorities for future research.

## 2 Materials and methods

### 2.1 Search strategy

A comprehensive systematic search was conducted in accordance with the 2020 Preferred Reporting Items for Systematic Reviews and Meta-Analyses (PRISMA) guidelines (Page et al., 2021). The protocol was prospectively registered in PROSPERO (registration number: CRD42025113695). Searches were performed across five databases — PubMed/MEDLINE, Scopus, Web of Science, Embase, and Cochrane Library — from database inception through August 2025.

The full PubMed search strategy was: (“aluminum” OR “aluminium”) AND (“autonomic nervous system” OR “heart rate variability” OR “baroreflex” OR “peripheral nerve” OR “enteric nervous system” OR “neurotoxicity” OR “nerve conduction velocity”). The use of both “aluminum” (US English) and “aluminum” (British English) was intentional to maximize retrieval across author conventions. Equivalent syntax adapted to each database’s controlled vocabulary is provided in **Supplementary Methods S2**.

Reference lists of all included studies and relevant narrative reviews identified during screening were hand-searched to capture eligible articles not indexed in the databases searched. Grey literature was not systematically searched; this is acknowledged as a limitation in Section 4.5. Duplicate records were removed using automated deduplication within each database platform, followed by manual cross-checking prior to abstract screening. The heterogeneity in study design and outcomes necessitated a mechanistic synthesis rather than a formal meta-analysis following the SWiM protocol (Campbell et al., 2020).

### 2.2 Eligibility criteria

Studies were eligible for inclusion if they: (i) involved human participants or experimental animal models with quantified or documented Al exposure via any route (dietary, occupational inhalation, intravenous, or oral gavage); (ii) reported at least one outcome pertaining to autonomic, peripheral, or enteric nervous system function, including HRV, baroreflex sensitivity (BRS), peripheral nerve conduction velocity, histopathological nerve assessment, or gut microbiota composition; and (iii) included a comparator group of unexposed or minimally exposed individuals or animals, or a pre-exposure baseline measurement.

Mechanistic and *in vitro* studies were also included if they directly examined molecular pathways — specifically, oxidative stress induction, calcium dysregulation, tight junction protein expression, neuroinflammatory signaling, or disruption of neurotransmitter/receptor systems implicated in autonomic or central regulatory control — that underlie the functional outcomes assessed in human and animal studies (see Section 2.1).

For the purposes of this review, human participants also include *ex vivo* models using human-derived biological material exposed to Al under controlled experimental conditions with an appropriate comparator arm.

Exclusion criteria were: (i) no relevant autonomic, peripheral, or enteric outcome measure; (ii) non-specific or unquantified Al exposure without verification; (iii) absence of a control or comparator group; (iv) editorials, conference abstracts, and narrative reviews without original data; and (v) studies available only as non-English language full texts without a verified translation. A full breakdown of exclusion reasons is provided in **Supplementary Table 1**.

### 2.3 Study selection and data extraction

All identified records were uploaded to Rayyan (Qatar Computing Research Institute) for deduplication and blinded screening. All four authors (TSG, PN, TS, and SB) independently screened all titles and abstracts against the eligibility criteria. Disagreements were resolved by discussion and consensus among the review team.

A total of 1938 records were identified across the five databases searched (see **Figure 1**). Following automated deduplication and removal of records marked ineligible by automation tools, 815 records were screened at the title/abstract level, of which 562 were excluded as not meeting the eligibility criteria in Section 2.2. Of the 253 full-text reports sought, 135 could not be retrieved (98 due to subscription-only access, 37 despite inter-library loan requests). Of the 118 full-text reports assessed for eligibility, 102 were excluded (**Supplementary Table 1**), yielding 16 studies from the original search. Zhou et al. (2015) was published in Chinese; the full text was translated (Google Translate to extract Methods-level detail for risk-of-bias assessment and **Supplementary Table 2**.

**Figure 1:**
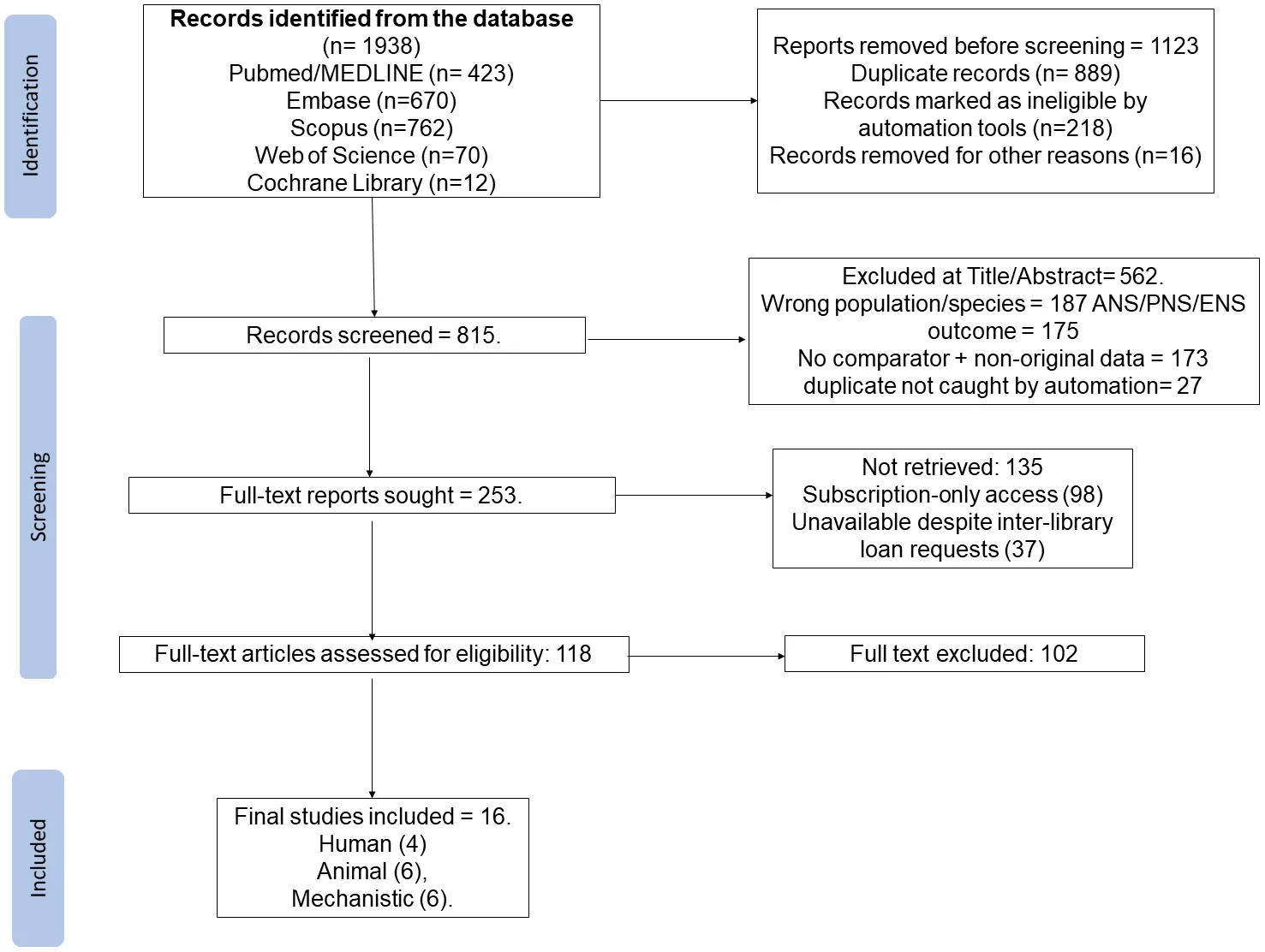
Study selection. PRISMA flow diagram shows the study selection process from PubMed, Embase, Scopus, Web of Science, and Cochrane Library. It lists records identified, screened, excluded, leading to 16 studies (human, animal, *in vitro*) for narrative synthesis.

Data from the 16 included studies were extracted independently by all four authors using a pre-piloted extraction form recording: first author and year; country; study design; population or animal model; sample size; Al exposure type, route, dose, and duration; comparator group; primary outcome measures; key quantitative findings; and risk of bias domains. Discrepancies were resolved by consensus.

For each included study, the analytical methodology for Al determination, biological matrix, analytical method, sample preparation, units, and reported validation parameters were extracted and compiled in **Supplementary Table 2**. Inter-rater agreement for title/abstract screening was calculated using Fleiss’ kappa (κ = 0.48; agreement). Agreement at the full-text stage was κ = 0.56 (Fleiss, 1971).

### 2.4 Synthesis approach

Given the substantial heterogeneity in study design, exposure type, and dose, and outcome measures across included studies, a formal meta-analysis was not feasible. Accordingly, a narrative synthesis with mechanistic mapping was employed, following the Synthesis Without Meta-analysis (SWiM) reporting guideline (Campbell et al., 2020). Evidence was organized into four physiological domains: autonomic nervous system (ANS) regulation; peripheral nervous system (PNS) and motor function; central structural and network connectivity; and enteric nervous system (ENS) and gut-brain axis.

It should be noted that the SWiM guideline was originally developed to synthesize heterogeneous evidence from intervention studies. Its application here to mechanistic and *in vitro* data extends beyond its original scope. We therefore supplemented the SWiM approach with a mechanistic pathway-mapping procedure adapted from the IARC framework for mechanistic evidence evaluation, which assigns weight to mechanistic data based on their strength, consistency, and biological plausibility across evidence tiers [International Agency for Research on Cancer (IARC) (2019)].

### 2.5 Risk of bias and certainty of evidence assessment

The methodological quality of included studies was assessed using validated tools appropriate to each study design: the Cochrane Risk of Bias 2.0 (RoB 2.0) tool for randomized controlled trials; the Newcastle-Ottawa Scale (NOS) for observational human studies; and the SYRCLE Risk of Bias tool for animal experiments. Mechanistic *in vitro* studies were assessed using a pragmatic framework evaluating five domains: exposure characterization, outcome validity, statistical reporting, biological replication, and selective reporting. Risk of bias (RoB) assessments were performed independently by all four authors (TSG, PN, TS, and SB), with discrepancies resolved by consensus.

Following individual study RoB assessment, the overall certainty of evidence for each outcome domain was evaluated using the Grading of Recommendations, Assessment, Development and Evaluation (GRADE) framework (**Table 1**). Although GRADE was originally developed for clinical intervention evidence, it was applied here — with acknowledged limitations — to provide a structured and transparent cross-tier certainty assessment incorporating risk of bias, inconsistency, indirectness, and imprecision. Its application to mechanistic and animal data was conservative and interpretive rather than strictly algorithmic, consistent with its adapted use in systematic reviews of environmental health.

**Table 1:** Certainty of evidence for aluminium-induced neurotoxicity across physiological domains.

Outcome domain	Evidence source	No. of studies	Risk of bias	Inconsistency	Indirectness	Imprecision	Publication bias	Overall certainty (GRADE)	Summary of findings
Autonomic dysfunction (HRV, Baroreflex Sensitivity)	Human (occupational + clinical)	2	Serious	Moderate	Serious	Serious	Possible	Low	Consistent reduction in HRV and impaired autonomic regulation; confounded by co-exposures (heat, noise)
	Animal studies	2	Moderate	Low	Moderate	Moderate	Unclear	Moderate	Reproducible impairment of baroreflex sensitivity and parasympathetic tone
	Mechanistic/*in vitro*	3	Low–Moderate	Low	Moderate	Moderate	Unclear	Moderate	Evidence of oxidative stress, calcium dysregulation affecting autonomic pathways
Peripheral nervous system dysfunction (nerve conduction, neuropathy)	Human (case reports)	1	Serious	Not assessable	Serious	Serious	Likely	Very Low	Single case report; myelopathy/neuropathy; not generalizable
	Animal studies	1	Moderate	Low	Moderate	Moderate	Unclear	Moderate	Direct Al deposition, inflammation, and oxidative stress demonstrated in sciatic nerve fibers, with associated mechanical allodynia; single study, not independently replicated
Central nervous system structural changes (MRI, connectivity)	Human	1	Low-Moderate	Not assessable	Moderate	Serious	Possible	Low	White matter damage and altered connectivity linked to aluminium exposure
	Mechanistic	3	Low-Moderate	Low	Moderate	Moderate	Unclear	Moderate	Blood-brain barrier disruption and neuroinflammation pathways demonstrated
Enteric nervous system & gut-brain axis	Animal studies	2	Moderate	Low	Moderate	Moderate	Unclear	Moderate	Gut dysbiosis and metal-homeostasis disruption consistent across two rodent studies
	Human (Ex-vivo)	1	Low moderate	Not assessable	Serious	Serious	Unclear	Low	Al-induced gut dysbiosis in human-derived colonic fermentation model
Mechanistic pathways (oxidative stress, Ca²^+^ dysregulation, NLRP3 activation)	*In vitro* + animal	7 (all 6 mechanistic + Hao/Zhu, minus double-count)	Low–Moderate	Low	Moderate	Low	Unclear	Moderate to High	Strong and consistent mechanistic evidence across models

Mechanistic and *in vitro* studies were evaluated using a pragmatic five-domain framework, with each domain rated as low, moderate, or high risk of bias according to pre-specified criteria. (1) Exposure characterization: low risk if the chemical form, concentration/dose, and duration of Al exposure were all explicitly reported and analytically verified; moderate risk if concentration and duration were reported but not analytically verified; high risk if exposure was described only qualitatively (e.g., ‘Al-treated’) without concentration or duration. (2) Outcome validity: low risk if outcome measures used a validated, previously characterized assay or antibody with reported specificity; moderate risk if a standard assay was used but validation was not reported; high risk if the assay or antibody was uncharacterized or novel without validation data. (3) Statistical reporting: low risk if exact p-values and effect sizes (or 95% confidence intervals) were reported for the outcome(s) relevant to this review; moderate risk if only significance thresholds (e.g., p < 0.05) were reported without effect sizes; high risk if statistical significance was asserted without any supporting statistics. (4) Biological replication: low risk if independent biological replicates (n >= 3) were explicitly stated and distinguished from technical replicates; moderate risk if replication was implied but not explicitly quantified; high risk if no replication information was provided. (5) Selective reporting: low risk if all outcomes stated in the methods were reported in the results; moderate risk if partial reporting occurred with a plausible non-selective explanation (e.g., data deferred to a supplementary table); high risk if outcomes stated in the methods were absent from the results without explanation. An overall study-level rating was assigned as the modal rating across the five domains, with any single high-risk domain capping the overall rating at moderate-high or high - an approach consistent with the conservative aggregation rule used for the RoB 2.0 and SYRCLE tools applied elsewhere in this review.

## 3 Results

The systematic search identified 16 primary studies meeting the inclusion criteria: human clinical and occupational studies (n=4), experimental animal models (n=6), and mechanistic/*in vitro* investigations (n=6). The synthesis is presented across four physiological domains (**Tables 2**–**4**). Although an initial large number of records (n = 1938) were identified across databases, 253 reports were sought for retrieval; 135 could not be accessed, resulting in 118 full-text articles assessed for eligibility, of which 102 were excluded with reasons indicated in **Supplementary Table 1**. This was primarily due to the stringent eligibility criteria applied, including the requirement for studies to directly evaluate Al exposure in relation to neurocardiovascular or autonomic outcomes, such as HRV, BRS, or related mechanistic pathways. A substantial proportion of studies were excluded due to a lack of relevant outcome measures, non-specific exposure assessment, or focus on unrelated toxicological endpoints. Additionally, a large number of records were removed during deduplication and automated screening processes, further reducing the pool of eligible studies. The relatively small number of included studies reflects the emerging nature of this research area, particularly in the area of integrative autonomic biomarkers.

**Table 2:** Human studies on aluminium neurotoxicity.

Author (year)	Population	Exposure	Outcomes	Key findings
He et al. (2003)	Al plant workers (n=200)	Occupational exposure (airborne Al dust)	Autonomic function tests	Reduced HRV, impaired parasympathetic activity.
Zhang et al. (2024)	Occupational workers	Chronic Al exposure	MRI (white matter, network connectivity)	White matter damage and disrupted functional networks were correlated with exposure levels.
Zhou et al. (2015)	Al smelting workers (n=110) vs. matched controls (n=110)	Occupational exposure (workplace air Al 6.45–7.02 mg/m³; serum/urinary Al elevated)	Autonomic function (Rmax : Rmin), cognitive (MMSE), neurobehavioral battery	Significantly reduced Rmax : Rmin (parasympathetic index) in exposed group; effect more pronounced with >10 years exposure vs. <5 years, indicating duration-dependent parasympathetic decline.
Verma and Sarkar (2019)	Case report (1 patient)	Chronic Al intoxication	Clinical exam, MRI	Myelopathy and neuropathy linked to Al overload.

Summary of clinical and epidemiological evidence linking aluminium exposure with neurological outcomes. Includes occupational cohorts and a case report. Major endpoints include heart rate variability and clinical features.

**Table 3:** Animal studies on aluminium neurotoxicity.

Author (year)	Model	Exposure and route	Outcomes	Key findings
Martinez et al. (2017)	Rats	AlCl_3_, oral, human dietary-equivalent dose (8.3 mg/kg/day), 60 days	Vascular function, blood pressure, oxidative stress markers	Al exposure promoted vascular dysfunction and increased blood pressure via concerted NAD(P)H oxidase and COX-2 activity.
Martinez et al. (2018)	Rats	AlCl_3_, oral, human dietary-equivalent dose (8.3 mg/kg/day), 60 days	Baroreflex sensitivity, autonomic regulation, peripheral function	Al exposure at human dietary levels significantly impaired baroreflex sensitivity and promoted peripheral dysfunction, linked to reduced nitric oxide bioavailability and increased sympathetic discharge.
Wang et al. (2022)	Rats	Chronic Al exposure	CNS (CaMKII/CREB), Gut microbiota	Altered hippocampal signaling; disrupted microbiota and SCFA production.
Shang et al. (2023)	Rats (5 timepoints: Day 7/14/30/60/90)	Al maltolate, 40 μmol/kg, intraperitoneal, every other day, with matched saline controls	Brain essential metal homeostasis (ICP-MS: Al, Zn, Cu, Fe, Cr, Mn, Co), gut microbiota composition (16S rRNA sequencing)	Progressive gut dysbiosis with increasing exposure duration (↓ Firmicutes, ↑ Proteobacteria and Actinobacteria); ten dysbiotic genera strongly correlated (r=0.70–0.90) with disrupted Fe, Zn, Mn, and Co levels in hippocampus, midbrain, and olfactory bulb.
Hao et al. (2023)	Mice	AlCl_3_	Neuroinflammation	NLRP3 inflammasome activation, neuronal pyroptosis.
Zhu et al. (2023)	Mice	Al	P2X7/NLRP3 pathway	Triggered neuroinflammation and glial activation.

Experimental findings from rodent models following aluminium exposure. Outcomes include vascular and autonomic dysfunction, peripheral nerve injury, hippocampal signaling changes, microbiota alterations, and neuroinflammatory pathways.

**Table 4:** Mechanistic and *in vitro* studies of aluminium neurotoxicity.

Author (year)	Model	Exposure	Outcomes	Key findings
Koenig and Jope (1987)	Cell culture	Al salts	Ca²^+^ influx	Inhibited voltage-dependent calcium channels.
Kaur and Gill (2005)	Rats	Chronic Al³^+^	Calcium homeostasis	Disturbed Ca²^+^ balance, excitotoxicity.
Julka et al. (1995)	Rats	Al³^+^	Cholinergic metabolism	Decreased acetylcholine turnover.
Kim et al. (2007)	Mice	Al³^+^	Dopamine receptor expression	Downregulated D2 receptor levels.
Yu et al. (2021)	Ex vivo human colonic fermentation (healthy adult donor stool)	Dietary-relevant Al, 86 mg/L, batch fermenters	Gut microbiota composition (16S rRNA sequencing)	Al altered human intestinal microbial community structure; co-supplementation with Lactobacillus plantarum (which bound Al at the cell surface) partially reversed Al-induced compositional changes.
De Sautu et al. (2018)	*In vitro*	Al³^+^	Ca²^+^ ATPases	Inhibited ATPase activity, impaired signaling.

Cellular, molecular, and ex vivo human studies demonstrate aluminium-induced disruptions in calcium signaling, neurotransmitter metabolism, dopamine receptor expression, ATPase activity, and gut microbiota composition, highlighting potential mechanistic pathways of neurotoxicity.

### 3.1 Autonomic nervous system regulation

Evidence across species suggests that Al exposure disrupts the autonomic control of cardiovascular function, primarily by reducing parasympathetic tone and impairing reflex sensitivity. In rat models, oral AlCl_3_ exposure at human dietary levels (8.3 mg/kg/day) for 60 days significantly impaired baroreflex sensitivity (Martinez et al., 2017, 2018). This impairment was linked to reduced nitric oxide (NO) bioavailability and increased sympathetic discharge (Nayak and Chatterjee, 2001a, 2001b; Martinez et al., 2017, 2018).

Occupational studies of Al plant workers (n=200) reported a significant reduction in HRV metrics, specifically the high-frequency (HF) component, indicating parasympathetic withdrawal (He et al., 2003). However, these findings are complicated by potential co-exposures such as heat and noise, which are known to influence autonomic tone. The consistent finding of reduced HRV and baroreflex dysfunction across both rodent models and human cohorts suggests that autonomic centers in the brainstem (e.g., nucleus tractus solitarius, NTS) are early targets of Al toxicity (He et al., 2003; Martinez et al., 2018).

This finding is corroborated by a larger occupational cohort among 110 Al smelting workers (batch preparation, electrolysis, and casting) compared with 110 age- and education-matched controls from the same facility. Workplace Al concentrations were significantly elevated (6.45–7.02 mg/m³ versus control levels, P<0.001), as were serum and urinary Al levels. Consistent with He et al. (2003), the Rmax: Rmin ratio (a validated index of parasympathetic cardiovascular control) was significantly lower in Al-exposed workers, with the effect more pronounced among workers with >10 years of exposure compared with those exposed for <5 years (Zhou et al., 2015). However, HRV is a non-specific biomarker and should be interpreted alongside other confounding exposure measurements.

### 3.2 Peripheral nervous system and motor function

Chronic Al exposure in rodents induces peripheral nerve pathology: Martinez et al. (2018) demonstrated direct Al deposition within sciatic nerve fibers, accompanied by nerve inflammation, oxidative stress, and mechanical allodynia, in rats exposed to human dietary-equivalent Al doses for 60 days (Martinez et al., 2018). This provides direct histological and functional evidence that peripheral nerves are a genuine target of Al accumulation, independent of central effects.

Human evidence is largely restricted to case reports of Al-induced myelopathy and peripheral neuropathy in patients with chronic renal failure or accidental high-level exposure (Verma and Sarkar, 2019). While these reports establish biological plausibility, the scarcity of large-scale epidemiological studies limits the generalizability of these findings. This clinical picture is consistent with rodent evidence of Al-induced oxidative damage and NLRP3-mediated neuroinflammatory injury (Zhou et al., 2022; Hao et al., 2023), and the similarity between rodent histopathology and human case reports indicates that Al-induced peripheral neurotoxicity is characterized by a dying-back pattern of axonal degeneration, likely triggered by disruption of the blood-nerve barrier (Zhu et al., 2024).

### 3.3 Central structural and network connectivity

Beyond cognitive scores, Al also impacts the CNS’s physical architecture and functional communication. These changes correlated with plasma and hair Al concentrations. *In vitro* studies suggest that these network disruptions are driven by Al’s ability to downregulate tight junction proteins (occludin, claudin-5), thereby increasing BBB permeability and subsequent neuroinflammation (Chen et al., 2008).

At the cellular level, Al-induced hippocampal neurodegeneration has been directly demonstrated in rodent models: chronic Al exposure causes neuronal death in the hippocampus, an effect attenuated by the antioxidant quercetin (Sharma et al., 2016), while more recent work shows that Al disrupts hippocampal neuronal development via dysregulation of m6A RNA methylation, an epigenetic mechanism controlling neuronal gene expression (Yang et al., 2024). Recent MRI studies in occupational cohorts demonstrated significant white matter damage and disrupted functional network connectivity in workers with chronic exposure (Zhang et al., 2024).

### 3.4 Enteric nervous system and gut-brain axis

Emerging evidence indicates that the gut-brain axis is a novel pathway for Al neurotoxicity. Experimental models show that Al ingestion induces gut dysbiosis, specifically reducing the abundance of Short-Chain Fatty Acid (SCFA)-producing bacteria. This dysbiosis is associated with increased intestinal permeability and the activation of pro-inflammatory pathways in the enteric plexuses, which may serve as a reservoir for systemic neuroinflammation (Wang et al., 2022; Claudino Dos Santos et al., 2023).

Yu et al. (2021) extended these findings using an *ex vivo* model of the human gut microbiota, in which fresh stool samples from healthy adult donors were exposed to a diet-relevant Al concentration (86 mg/L) in batches of colonic fermenters. Al exposure altered the structure of the human intestinal microbial community. Co-supplementation with Lactobacillus plantarum, which bound Al directly at the cell surface, partially reversed these Al-induced compositional changes, suggesting a potential probiotic-based mitigation strategy for Al-induced gut dysbiosis in humans (Yu et al., 2021).

A more recent rodent study corroborates and extends these findings, using a five-timepoint exposure design (Al-maltolate, 40 μmol/kg i.p., every other day for 7–90 days) with matched saline controls. Shang et al. (2023) found progressive gut dysbiosis with increasing Al-exposure durationby Day 90. Notably, they showed the greatest dysbiotic shift correlated strongly (r = 0.70–0.90) with Al-induced disruption of essential metal homeostasis (Fe, Zn, Mn, Co) in the hippocampus, midbrain, and olfactory bulb (Shang et al., 2023). This study hints at gut microbiota disturbance and brain metal dyshomeostasis may be mechanistically linked components of the same Al-toxicity pathway.

### 3.5 RoB of human studies

The methodological quality of the included studies was variable. Most occupational cohorts (He et al., 2003; Zhang et al., 2024) demonstrated reasonably robust selection and exposure characterization but had limited control for confounders, including co-exposures and lifestyle factors. Outcome assessment was generally appropriate, although evaluator blinding and repeated-measures analyses were seldom reported. Overall, these studies were judged to be at moderate risk of bias. Zhou et al. (2015) study was judged to be at moderate RoB, reflecting adequate exposure characterization and a matched comparator group, though control for comorbidity and lifestyle factors could not be fully confirmed from the translated text (Zhou et al., 2015). Verma and Sarkar (2019), study which is a single case report, was considered high risk due to its inherently subjective, non-comparative design (Verma and Sarkar, 2019). The traffic light signaling representation of the RoB is shown in **Figures 2A, B**.

**Figure 2:**
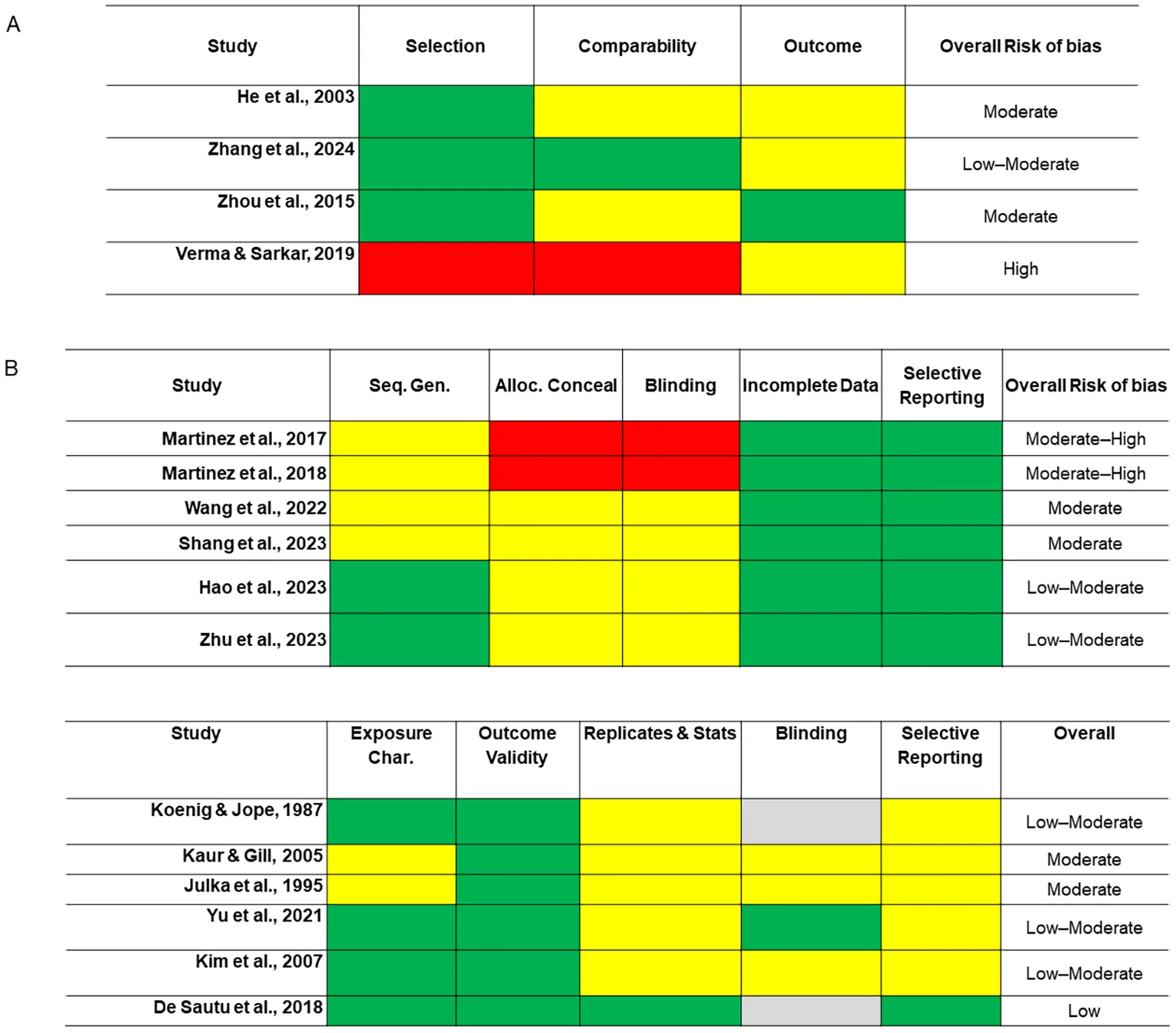
**(A)** Risk of bias in human studies. Risk of bias in human studies was assessed with the Newcastle-Ottawa Scale (NOS). The chart shows quality across selection, comparability, and outcome domains. Concerns include not controlling for environmental confounders like noise and heat stress in aluminium smelting facilities. **(B)** Risk of bias in animal studies. Risk of bias in experimental and animal studies was assessed with the SYRCLE tool, covering sequence generation, allocation concealment, housing, and blinded outcome assessment. Most studies had strong validity, though reporting on random housing and investigator blinding was inconsistent.

### 3.6 RoB of animal studies

Animal studies frequently failed to report random sequence generation, allocation concealment, and investigator blinding, reflecting common limitations in the preclinical literature. Nevertheless, outcome reporting and attrition control were generally adequate. Earlier experiments raised greater concerns, while more recent work (Hao et al., 2023; Zhu et al., 2024) demonstrated better methodological transparency. Overall, most animal studies had moderate-to-high RoB, with only the most recent reports approaching low-to-moderate risk (**Figure 2B**).

### 3.7 ROB of mechanistic evidence

Mechanistic *in vitro* studies were evaluated pragmatically for exposure characterization, outcome validity, and reproducibility. While most provided clear exposure descriptions and valid outcome assays, statistical reporting and replication were inconsistently documented. These studies were therefore judged to be at low to moderate risk of bias (**Table 1**).

Taken together, the RoB assessment indicates that although the available evidence consistently points to systemic neurotoxic effects of Al, the overall certainty is tempered by methodological shortcomings, particularly in earlier animal studies and observational human data. This underscores the need for more rigorously designed epidemiological investigations and standardized preclinical protocols. However, across evidence tiers, a convergent pattern emerges. Human occupational studies report reduced HRV and altered brain connectivity, suggesting early autonomic involvement, though confounding and exposure misclassification limit causal inference. Animal studies consistently demonstrate impaired baroreflex sensitivity, vascular dysfunction, and slowed peripheral nerve conduction following Al exposure. These functional changes align with mechanistic findings of OS, mitochondrial dysfunction, calcium dyshomeostasis, and neuroinflammation. However, the strength of evidence varies substantially, with animal and mechanistic data providing stronger support than current human studies.

## 4 Discussion

The integrated analysis of evidence across human, animal, and mechanistic studies reveals a consistent pattern: Al exerts neurotoxic effects that extend substantially beyond the CNS. While the mechanistic foundation — involving OS, neurovascular barrier disruption, calcium dyshomeostasis, and neuroinflammation — is well supported across all evidence tiers, the clinical evidence is strongest for autonomic dysfunction, as reflected by changes in HRV and BRS. Peripheral and enteric nervous system findings remain biologically plausible but hypothesis-generating in humans.

Importantly, not all evidence points in the same direction. He et al. (2003) and Zhou et al. (2015) reported reduced parasympathetic HRV indices in Al-exposed workers, yet effect sizes varied by exposure duration and were attenuated after accounting for demographic covariates (He et al., 2003; Zhou et al., 2015). This variability warrants engagement rather than dismissal and underscores the need for standardized, longitudinal exposure assessment (Section 4.4).

The overall certainty of evidence, assessed using GRADE, was rated low for human studies of autonomic dysfunction (owing to serious risk of bias, indirectness, and imprecision) and moderate for animal and mechanistic studies. Evidence for peripheral and enteric nervous system involvement in humans should be considered very low certainty. These ratings reflect the current state of the field; the scarcity of well-designed human studies targeting integrative autonomic endpoints in Al-exposed populations is itself a major identified gap.

### 4.1 The barrier-stress axis: a proposed mechanistic model

The assessment of HRV and BRS serves as a critical window into the operational integrity of the autonomic nervous system (ANS). Because Al exposure and neurodegenerative processes often disrupt autonomic regulatory centers before gross structural damage is visible, these metrics function as potential candidate as functional biomarkers (He et al., 2003; Zhou et al., 2015).

HRV measures naturally occurring beat-to-beat variations in heart rate, providing a non-invasive quantitative assessment of the balance between sympathetic and parasympathetic activity. In occupational settings, workers exposed to metals such as Al, lead, and zinc show alterations in R-R interval variability. These changes often correlate with peripheral nerve conduction abnormalities, suggesting that HRV can detect subclinical neurotoxicity (Brodeur et al., 1999; He et al., 2003).

HRV indexes are significantly altered in patients with dementia, including Alzheimer’s Disease (AD) and Vascular Dementia. While some studies suggest that autonomic function may appear normal in early stages, systematic reviews indicate a general decline in HRV metrics as cognitive impairment progresses (Allan et al., 2005, 2006). Chronic Al exposure has been shown to induce peripheral dysfunction and promote vascular dysfunction at human dietary levels. By monitoring HRV, clinicians may identify the early silent phase of Al-induced autonomic strain (He et al., 2003; Martinez et al., 2018; Zhou et al., 2022).

The baroreflex is a fundamental homeostatic mechanism that regulates blood pressure via the ANS. Al exposure interferes with this control through several pathways. Al promotes the concerted action of NADPH oxidase and COX-2, thereby increasing blood pressure and causing vascular dysfunction. This oxidative stress can impair the baroreceptors’ ability to respond to pressure changes (Kumar et al., 2009; Bondy, 2016; Martinez et al., 2017). The cardiac impact includes experimental models that show that Al exposure can increase heart rate without a corresponding elevation in cardiac output, suggesting a decoupling of standard cardiovascular regulatory mechanisms (He et al., 2003; Krewski et al., 2007; Chen et al., 2008; Martinez et al., 2018). Since Al accumulates in tissues and affects calcium signaling (Ca^+2^-ATPases) and neurotransmitter systems (GABA, glutamate, and dopamine), the baroreflex serves as an integrated physiological readout of these complex molecular disruptions (Exley and Burgess, 1996; Nayak and Chatterjee, 2001b; Kaur and Gill, 2005; Kawahara and Kato-Negishi, 2011; Bondy, 2016).

The utility of these biomarkers is grounded in the specific ways Al disrupts the gut-heart-brain axis via neuroinflammation. Al activates the NLRP3 inflammasome, leading to neuroinflammation that can damage autonomic control centers in the brain (Hao et al., 2023; Zhu et al., 2023). Further, by inhibiting voltage-dependent calcium influx and disrupting intracellular Ca^+2^ homeostasis, Al impairs the signaling required for smooth autonomic responses (Kawahara and Kato-Negishi, 2011; Exley, 2013). Dietary Al disrupts gut microbiota and intestinal barriers (Wang et al., 2022; Zhu et al., 2024). Since the vagus nerve facilitates crosstalk between the gut and the brain, changes in HRV may reflect early intestinal and systemic inflammation induced by Al (Brodeur et al., 1999; Yu et al., 2021).

The clinical takeaway from this synthesis is the identification of HRV and BRS as potential early-warning systems. In occupational health, the current gold standard is the plasma Al levels, which often fail to reflect the true toxic burden, as Al is a bone-seeking element that rapidly sequesters into deep tissue compartments (Priest, 2004; Krewski et al., 2007).

Our analysis suggests that the ANS may serve as an early functional indicator via a two-step “Barrier-Stress” axis. Barrier disruption occurs when Al downregulates tight junction proteins, specifically occludin and claudin-5, thereby disrupting the integrity of the Blood-Brain Barrier (BBB) and the Blood-Nerve Barrier (BNB) (Hao et al., 2022, 2023). This compromise allows Al to accumulate in the NTS, the brainstem’s primary regulatory center for cardiovascular reflexes (Martinez et al., 2017, 2018). However, current human evidence remains limited.

### 4.2 Mechanistic convergence

We propose that Al’s neurotoxicity is a two-step process, including barrier Disruption, in which Al disrupts the claudin/occludin integrity of the blood-brain and blood-nerve barriers (Chen et al., 2008; Hao et al., 2022). Once these barriers are compromised, Al can initiate a cascade of oxidative stress (OS) and inflammatory signaling characterized by lipid peroxidation and the release of pro-inflammatory cytokines such as TNF-α and IL-6 (Sharma et al., 2016; Zhou et al., 2022). Experimental evidence also indicates that Al activates the NLRP3 inflammasome pathway, leading to neuroinflammation and neuronal injury within the central nervous system (Hao et al., 2022).

Al-induced disruption extends to neurotransmitter receptor systems more broadly: Al exposure has been shown to decrease dopamine D1 and D2 receptor expression in mouse brain (Kim et al., 2007), in addition to its documented effects on GABAergic and glutamatergic signaling (Nayak and Chatterjee, 2001b). Since central dopaminergic pathways contribute to autonomic outflow regulation via hypothalamic and brainstem projections, this receptor-level disruption may represent an additional, currently unexplored route by which Al compromises autonomic control alongside the barrier-disruption and oxidative-stress mechanisms already discussed.

In addition, emerging evidence suggests that toxic metals can influence gut microbial composition and intestinal barrier integrity, thereby affecting gut-brain communication pathways (Zhu et al., 2024). Taken together, these findings may explain the multisystem manifestations of Al toxicity, in which a common molecular mechanism produces organ-specific outcomes, such as gut dysbiosis within the enteric nervous system and impaired neural conduction within peripheral nerves.

### 4.3 Clinical utility of HRV and baroreflex sensitivity as early functional biomarkers

The baroreflex represents a highly integrated physiological mechanism that maintains short-term blood pressure homeostasis through coordinated interactions between the central nervous system, autonomic pathways, and peripheral cardiovascular effectors (Swenne, 2013). Because it reflects the dynamic balance between sympathetic and parasympathetic activity, BRS and its surrogate marker, HRV, are increasingly recognized as sensitive indicators of subclinical autonomic dysfunction (La Rovere et al., 2008). Unlike isolated cardiovascular parameters such as heart rate or blood pressure, the baroreflex provides a systems-level readout of neurocardiovascular regulation, making it particularly suited to detect early and subtle disturbances across multiple physiological domains (Parati et al., 2000).

Al exposure is known to disrupt several core mechanisms underlying baroreflex function. Experimental studies demonstrate that Al induces OS and inflammatory signaling pathways, thereby impairing vascular reactivity and autonomic regulation (Sharma et al., 2016; Zhou et al., 2022). In addition, Al disrupts calcium homeostasis by altering Ca²^+^-ATPase and voltage-gated calcium channel activities, thereby impairing the intracellular signaling required for effective autonomic neurotransmission (Kaur and Gill, 2005). Al has also been shown to alter key neurotransmitter systems, including glutamatergic and GABAergic pathways, which are essential for central autonomic integration (Nayak and Chatterjee, 2001b). Furthermore, activation of neuroinflammatory pathways, including the NLRP3 inflammasome, may directly affect brainstem autonomic control centers, further compromising baroreflex function (Hao et al., 2023). Collectively, these mechanisms suggest that Al toxicity converges on pathways critical to maintaining baroreflex integrity.

It is important to note that neither HRV nor BRS is specific to Al toxicity. Both are influenced by physical activity, sleep quality, psychological stress, caffeine intake, ambient temperature, noise exposure, shift-work status, certain medication classes (e.g., beta-blockers, anticholinergics), and body mass index. The occupational cohort studies included in this review (He et al., 2003; Zhang et al., 2024) controlled for standard demographic and clinical confounders — age, sex, education, income, smoking, and comorbid cardiovascular or neuropsychiatric disease. However, neither reported controlling for workplace heat or noise, despite both being conducted in Al electrolytic/smelting environments where such co-exposures are well documented (Section 4.4). Consequently, while confounding by common demographic and health factors is unlikely to fully explain the observed HRV/BRS reductions, residual confounding by occupational heat and noise exposure cannot be excluded, and future validation studies should measure and adjust for these occupational co-exposures.

From a systems perspective, the baroreflex also serves as a functional interface within the gut-brain-heart axis. Emerging evidence indicates that toxic metals can alter gut microbiota composition and intestinal barrier integrity, thereby influencing vagally mediated communication between the gut and central autonomic networks (Zhu et al., 2024). Given the central role of the vagus nerve in modulating both cardiovascular and gastrointestinal function, disruptions along this axis may be reflected in measurable changes in HRV and BRS. Importantly, these alterations may occur prior to overt clinical manifestations, highlighting the potential utility of BRS and HRV as early, non-invasive biomarkers of Al-induced systemic toxicity.

Taken together, these observations support the concept that BRS is not merely a cardiovascular parameter but an integrative biomarker that captures the cumulative impact of OS, calcium dysregulation, neuroinflammation, and disturbances of the gut–brain axis associated with Al exposure, as illustrated in **Figure 3**.

**Figure 3:**
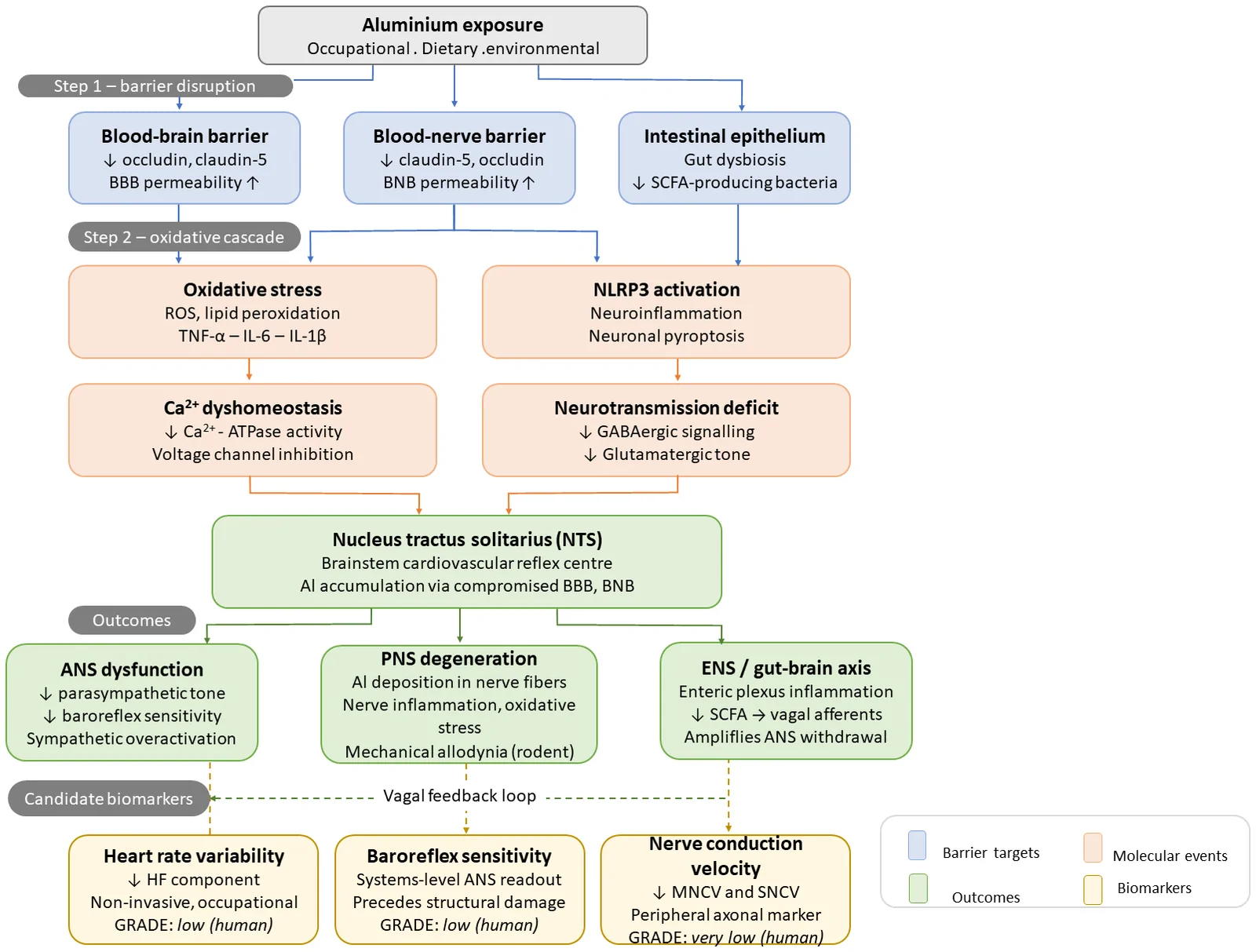
Proposed mechanistic pathway for the impact of Al on the autonomic nervous system. The proposed aluminium toxicity pathways: (i) Intestinal Barrier, where chronic Al exposure disrupts proteins like occludin and claudin-5, causing gut dysbiosis and permeability; (ii) Autonomic Signaling, where gut-brain axis disruption triggers vagal signaling and activates NLRP3 inflammasome, leading to parasympathetic withdrawal and lower Heart Rate Variability (HRV); (iii) Peripheral Impact, with Al accumulating in nerves, causing oxidative stress, inflammation, and Ca²^+^-ATPase inhibition, leading to mechanical allodynia in rodents, indicating nerve damage.

### 4.4 Methodological strategies to address confounders in the future

The recommendation to use longitudinal designs to capture “subclinical” markers, such as HRV, is supported by current occupational health evaluations. Recent toxicological assessments for Al-exposed workers have shifted focus to subclinical effects, establishing a biological tolerance (BAT) value of 50 µg/g creatinine based on subtle neuropsychological and physiological changes (Klotz et al., 2017). Longitudinal data are critical because Al has a highly variable biological half-life, ranging from a few hours to several years depending on the storage compartment (e.g., bones or lungs), which can mask early autonomic signals in cross-sectional studies (Klotz et al., 2017).

Workplace heat exposure is a major challenge, placing manual workers at high risk of heat stress (Xiang et al., 2014). We hypothesize that, in Al smelting environments where process-generated heat imposes cardiovascular strain (Xiang et al., 2014), pre-existing Al-induced impairment of baroreflex-mediated parasympathetic control (Martinez et al., 2017, 2018) could further reduce workers’ physiological resilience, a hypothesis that has not, to our knowledge, been directly tested (Xiang et al., 2014).

Chronic occupational noise is a known risk factor for noise-induced hearing loss and autonomic arousal (Lie et al., 2016). Combined occupational exposure to metal particulates and noise has been shown to produce a greater imbalance in sympathetic-parasympathetic HRV parameters than either exposure alone, suggesting a synergistic effect on autonomic regulation that may compound Al-induced autonomic strain (Lucas et al., 2022). Studies in Al smelter cohorts have linked chronic exposure to combustion products (like PAHs) with increased ischemic heart disease mortality, emphasizing the need to account for cumulative, multi-exposure environments (Friesen et al., 2010).

Moreover, there is a critical need for Longitudinal Human Cohorts that track autonomic function (HRV) and peripheral nerve health in workers from the start of their exposure. Given emerging data on Al-induced gut dysbiosis (Wang et al., 2022), researchers should investigate whether SCFA supplementation can mitigate autonomic withdrawal in Al-exposed models. Future work must establish clinical cutoff values for Al-induced autonomic changes to distinguish them from general lifestyle-induced stress.

### 4.5 Limitations

There are many restrictions on this review. First, co-exposures, lifestyle variables, and occupational stresses may confound the majority of cross-sectional human research. Second, the evaluation of Al exposure is inconsistent and sometimes indirect. Third, direct extrapolation to humans is limited by the significant variations in dosage, duration, and exposure mode observed in animal research. A structured review of analytical methodology across the included studies (**Supplementary Table 2**) found that none reported standard validation parameters (LOD, LOQ, recovery, or certified reference materials) for Al quantification, compounding the confounding issues discussed in Section 4.3.1.

The relatively small number of included studies (n = 16) represents a limitation of the present review. Moreover, non-retrieval of 135 full-text records, predominantly due to subscription-only access (n=98), represents a potential source of RoB due to missing evidence (Higgins et al., 2023). While the direction and magnitude of such bias cannot be determined without the missing data themselves, empirical evidence on its impact on meta-analytic and narrative synthesis conclusions remains mixed (Schmucker et al., 2017).

However, this likely reflects the limited availability of studies specifically examining Al exposure in relation to integrative autonomic outcomes, such as BRS and HRV. Furthermore, the inclusion of heterogeneous study designs (human, animal, and mechanistic) was necessary to provide a comprehensive understanding of the underlying biological mechanisms, but this may limit the generalizability of the findings. Future well-designed human studies focusing on standardized autonomic endpoints are required to strengthen the evidence base. Despite the limited number of eligible studies, there was consistent convergence of evidence from experimental and mechanistic models supporting Al-induced autonomic and neuroinflammatory dysfunction.

## 5 Conclusion

Evidence indicates that Al exerts neurotoxic effects across multiple systems, not just the CNS. Mechanistically, Al induces OS, mitochondrial dysfunction, calcium dyshomeostasis, and neurotransmitter imbalance, and activates glial inflammation and disrupts the BBB (Koenig and Jope, 1987; Julka et al., 1995; Exley, 2013; De Sautu et al., 2018; Hao et al., 2022; Zhu et al., 2023). These changes have been linked to cognitive deficits, autonomic dysfunction, slowed peripheral conduction, and alterations in the gut-brain axis (He et al., 2003; Zhou et al., 2015; Wang et al., 2022).

Taken together, the findings suggest that Al neurotoxicity may contribute to a multisystem process, with early involvement of autonomic and peripheral domains. HRV and BRS represent promising candidate markers for early autonomic involvement following Al exposure (He et al., 2003; Zhou et al., 2015; Wang et al., 2022). However, current evidence is insufficient to support their use as established screening tools, and many animal studies lack rigorous methodological controls. Prospective human studies with standardized exposure assessment are required before clinical application.

In conclusion, Al’s neurotoxic profile extends beyond the CNS, warranting longitudinal human studies with standardized endpoints. At present, these findings should be interpreted as hypothesis-generating rather than definitive evidence of early diagnostic markers in humans. Further investigation of early autonomic and peripheral changes may help inform future preventive and therapeutic research strategies.

## Supplementary material

Supplementary Table 1 Risk-of-bias criteria for mechanistic and *in vitro* studies. Overall study rating = modal rating across the five domains; any single high-risk domain caps the overall rating at moderate-high or high.

Supplementary Table 2 Aluminium exposure assessment and analytical methodology across included studies. Summary of the biological matrix, analytical method, sample preparation protocol, species measured, units of measurement, reported analytical validation parameters, and exposure window for aluminium determination across all 16 studies included in this systematic review. This table was compiled in response to reviewer comments requesting a detailed account of exposure-assessment methodology across the evidence base. Where a study administered aluminium experimentally without independently verifying tissue, plasma, or cellular Al concentrations, this is noted explicitly. Validation parameters refer to limit of detection (LOD), limit of quantification (LOQ), recovery percentage, precision (relative standard deviation, RSD), and use of certified reference materials (CRMs); “None reported” indicates that none of these parameters were stated in the source publication’s Methods section.
