# Supplementary material for: Aluminium neurotoxicity beyond the brain: a mechanistic systematic review of autonomic and peripheral dysfunction
Source: Front Physiol. 2026 Sep 16;17:1891526. doi: 10.3389/fphys.2026.1891526 (PMC13624941; doi:10.3389/fphys.2026.1891526)
Supplement: Supplementary file 1 [file DataSheet1.doc]

Methods S2. Detailed Search Strategy

**Pubmed**

("aluminium"[Title/Abstract] OR "aluminum"[Title/Abstract]

OR "aluminium exposure"[Title/Abstract] OR "aluminum toxicity"[Title/Abstract])

AND

("autonomic nervous system"[MeSH Terms] OR "autonomic nervous system"[Title/Abstract]

OR "heart rate variability"[Title/Abstract]

OR "baroreflex sensitivity"[Title/Abstract]

OR "peripheral nerve"[Title/Abstract]

OR "peripheral neuropathy"[Title/Abstract]

OR "enteric nervous system"[Title/Abstract]

OR "gut-brain axis"[Title/Abstract]

OR "neurotoxicity"[Title/Abstract])

AND

("humans"[MeSH Terms] OR "animals"[MeSH Terms])

Filters: English, from database inception to August 2025

**Scopus**

(TITLE	(aluminium OR aluminum OR "aluminium exposure" OR "aluminum toxicity"))

AND

(TITLE ("autonomic nervous system" OR "heart rate variability"

OR "baroreflex sensitivity"

OR "peripheral nerve" OR "peripheral neuropathy"

OR "enteric nervous system"

OR "gut-brain axis"

OR "neurotoxicity"))

AND

(LIMIT-TO (LANGUAGE, "English"))

**Web of Science Core Collection**

TS=(aluminium OR aluminum OR "aluminium exposure" OR "aluminum toxicity")

AND

TS=("autonomic nervous system" OR "heart rate variability"

OR "baroreflex sensitivity"

OR "peripheral nerve" OR "peripheral neuropathy"

OR "enteric nervous system"

OR "gut-brain axis"

OR "neurotoxicity")

Refined by: Languages = English

Timespan: All years to August 2025

**Embase**

('aluminium'/exp OR 'aluminum'/exp OR 'aluminium exposure' OR 'aluminum toxicity')

AND

('autonomic nervous system'/exp OR 'heart rate variability'

OR 'baroreflex sensitivity'

OR 'peripheral nerve'/exp

OR 'peripheral neuropathy'

OR 'enteric nervous system'

OR 'gut brain axis'

OR 'neurotoxicity'/exp)

AND

[english]/lim

AND

[humans]/lim OR [animals]/lim

**Cochrane Library**

(aluminium OR aluminum OR "aluminium exposure" OR "aluminum toxicity")

AND

("autonomic nervous system" OR "heart rate variability"

OR "baroreflex sensitivity"

OR "peripheral nerve" OR "peripheral neuropathy"

OR "enteric nervous system"

OR "gut-brain axis"

OR "neurotoxicity")

Table S1. Reasons for Non-Retrieval and Exclusion During Full-Text Screening

Section A: Reasons full-text reports could not be retrieved (n = 135)

| **Reason** | **n** | **Description** |
| --- | --- | --- |
| Subscription-only access | 98 | Full text inaccessible through institutional subscriptions |
| Unavailable despite inter-library loan requests | 37 | No full text located despite formal ILL requests |
| Total | 135 |  |

Section B — Reasons for exclusion among full-text reports assessed for eligibility (n = 102)

| **Reason** | **n** | **Description** |
| --- | --- | --- |
| Conference abstracts, no original data. | 41 | records were identified as conference abstracts at full-text review; excluded per a priori criterion (Section 2.2, criterion iv) |
| Irrelevant outcomes | 25 | No autonomic, peripheral, or enteric nervous system outcome measure (e.g., cognition-only, bone metabolism, renal toxicity, or CNS-only endpoints such as amyloid/secretase pathways) |
| Non-specific aluminium exposure | 12 | Exposure not clearly quantified, or Al not the primary/stratified exposure in a mixed-metal study |
| No control/comparator group | 10 | No unexposed comparator or pre-exposure baseline available |
| Inadequate or non-validated outcome measures | 8 | Outcomes not directly measurable or lacking methodological validation |
| Review articles/editorials/commentaries | 3 | Narrative reviews or opinion pieces without original data |
| No documented aluminium exposure | 2 | General dementia/Alzheimer's disease literature with no quantified or reported Al exposure (Section 2.2, criterion i) |
| Non-English, no verified translation | 1 | Full text unavailable in English or via verified translation (Section 2.2, criterion v) |
| Total | 102 |  |
